# Liver graft hyperperfusion in the early postoperative period promotes hepatic regeneration 2 weeks after living donor liver transplantation: A prospective observational cohort study

**DOI:** 10.1097/MD.0000000000005404

**Published:** 2016-11-18

**Authors:** Sung Hye Byun, Hae Soo Yang, Jong Hae Kim

**Affiliations:** Department of Anesthesiology and Pain Medicine, School of Medicine, Catholic University of Daegu, Daegu, Republic of Korea.

**Keywords:** hemodynamics, liver regeneration, liver transplantation, living donors

## Abstract

Hepatic regeneration is essential to meet the metabolic demands of partial liver grafts following living donor liver transplantation (LDLT). Hepatic regeneration is promoted by portal hyperperfusion of partial grafts, which produces shear stress on the sinusoidal endothelium. Hepatic regeneration is difficult to assess within the first 2 weeks after LDLT as the size of liver graft could be overestimated in the presence of postsurgical graft edema. In this study, we evaluated the effects of graft hyperperfusion on the rate of hepatic regeneration 2 weeks after LDLT by measuring hepatic hemodynamic parameters. Thirty-six patients undergoing LDLT were enrolled in this study. Hepatic hemodynamic parameters including peak portal venous flow velocity (PVV) were measured using spectral Doppler ultrasonography on postoperative day 1. Subsequently, we calculated the ratio of each velocity to 100 g of the initial graft weight (GW) obtained immediately after graft retrieval on the day of LDLT. Ratios of GW to recipient weight (GRWR) and to standard liver volume (GW/SLV) were also obtained. The hepatic regeneration rate was defined as the ratio of the regenerated volume measured using computed tomographic volumetry at postoperative week 2 to the initial GW. Correlations of the hemodynamic parameters, GRWR, and GW/SLV with the hepatic regeneration rate were assessed using a linear regression analysis. The liver grafts regenerated to approximately 1.7 times their initial GW (1.7 ± 0.3 [mean ± standard deviation]). PVV/100 g of GW (*r*^2^ = 0.224, β_1_ [slope coefficient] = 2.105, *P* = 0.004) and velocities of the hepatic artery and vein per 100 g of GW positively correlated with the hepatic regeneration rate, whereas GRWR (*r*^2^ = 0.407, β_1_ = –81.149, *P* < 0.001) and GW/SLV (*r*^2^ = 0.541, β_1_ = –2.184, *P* < 0.001) negatively correlated with the hepatic regeneration rate. Graft hyperperfusion demonstrated by increased hepatic vascular velocities and a small-sized graft in the early postoperative period contributes to hepatic regeneration 2 weeks after LDLT.

## 1 Introduction

Living donor liver transplantation (LDLT) is a well-established treatment for end-stage liver disease, especially in Asian countries with a shortage of cadaveric donors. Unlike the whole liver allografts which are transplanted from deceased donors, LDLT uses a smaller partial liver graft and therefore, it is important to balance the requirements for maintaining adequate remnant liver volume in the donor with those for ensuring appropriate graft volume in the recipient, in order to meet the metabolic demands of the recipient and ensure the safety of both patients.^[1]^ Fortunately, the issue of a small-sized liver graft can be resolved by rapid regeneration of liver volume, which generally restores the graft to its standard volume within 2 weeks post-LDLT.^[2–4]^ After graft implantation, liver cells within the graft, including the Kupffer cells and sinusoidal endothelial cells, are exposed to a large amount of portal venous blood flow (PVF) and the shear stress generated by this PVF on endothelial surface stimulates hepatic regeneration.^[5–7]^ Several previous studies have demonstrated that portal venous hemodynamics representing the shear stress have a major impact on hepatic regeneration by showing the correlations between PVF or portal venous flow velocity assessed at different postoperative time points and the degree of graft regeneration.^[2,8–10]^ The most appropriate time for evaluating graft regeneration in the early postoperative period appears to be during the 2nd postoperative week. Although a substantial amount of liver regeneration occurs within the first 2 postoperative weeks followed by a plateau within 1 to 2 months after LDLT,^[2,3]^ a large amount of graft edema also occurs secondary to portal hyperperfusion of partial grafts and thus, precludes an accurate assessment of hepatic regeneration within 2 weeks after LDLT.^[11]^

Therefore, we investigated the effects of graft hyperperfusion during the early postoperative period on hepatic regeneration 2 weeks after LDLT by assessing the correlations of hepatic hemodynamic parameters per initial graft weight (GW), which were measured by spectral Doppler ultrasonography on postoperative day (POD) 1; graft-to-recipient weight ratio (GRWR); and GW to standard liver volume ratio (GW/SLV), which were obtained immediately after the graft retrieval, with the hepatic regeneration rate at 2 weeks after LDLT.

## 2 Materials and methods

### 2.1 Study population

After obtaining study approval from Daegu Catholic University Medical Center Institutional Review Board and written informed consent from the patients, this prospective observational cohort study was conducted using 36 consecutive patients undergoing LDLT (31 right-lobe and 5 left-lobe grafts) from March 2013 to January 2014. Patients were included if they had liver cirrhosis with or without concurrent hepatocellular carcinoma. Patients were excluded if they underwent LDLT due to acute liver failure or if they required repeat surgical interventions within the study period; if they failed to be weaned from mechanical ventilation within 24 hours of arrival to the surgical intensive care unit after surgery; if they had vascular complications of the portal vein, hepatic artery, or hepatic vein; or if they had missing data.

### 2.2 Anesthetic management

Anesthesia was induced using 1.5 to 2 mg/kg of propofol in combination with 0.01 to 0.05 μg/kg/min of remifentanil. Endotracheal intubation was facilitated with 1 mg/kg of rocuronium. Anesthesia was maintained using desflurane in an air/O_2_ mixture with continuous infusion of remifentanil. Desflurane and remifentanil were regulated to maintain a bispectral index value between 40 and 60 along with mean arterial pressure and heart rate within 25% of preinduction values. Rocuronium was continuously infused at a rate of 1 mg/kg/h for neuromuscular blockade during the surgery. All patients were mechanically ventilated without positive end expiratory pressure at a constant tidal volume of 6 to 10 mL/kg. The respiratory rate and fraction of inspired oxygen were adjusted to maintain arterial carbon dioxide and oxygen tensions between 35 to 40 mm Hg and 150 to 200 mm Hg, respectively. Prostaglandin E_1_ was continuously administered from the beginning of anastomosis of hepatic artery at a rate of 0.01 μg/kg/min. After surgery, all patients were admitted to a surgical intensive care unit and extubated within 24 hours of arrival.

### 2.3 Surgical technique

When the right lobe of a donor was used, a single and large hepatic venous outflow was constructed by one-orifice venoplasty of the graft's right hepatic vein and reconstructed middle hepatic vein.^[12]^ Following full mobilization of the native liver and its vascular exclusion, total hepatectomy was performed with preservation of the inferior vena cava by selective clamping of the right hepatic vein and, middle and left hepatic vein trunk without venovenous bypass. One of the orifices of the middle and left hepatic vein trunk and the right hepatic vein was closed according to the type of liver graft and the remaining orifice was anastomosed to the donor hepatic vein under partial clamping of the inferior vena cava. After the end-to-end anastomosis of the portal vein, the liver graft was reperfused. Finally, the hepatic artery and bile duct were reconstructed and the abdominal wall was closed.

### 2.4 Measurement of hepatic hemodynamic parameters

**Figure 1:**
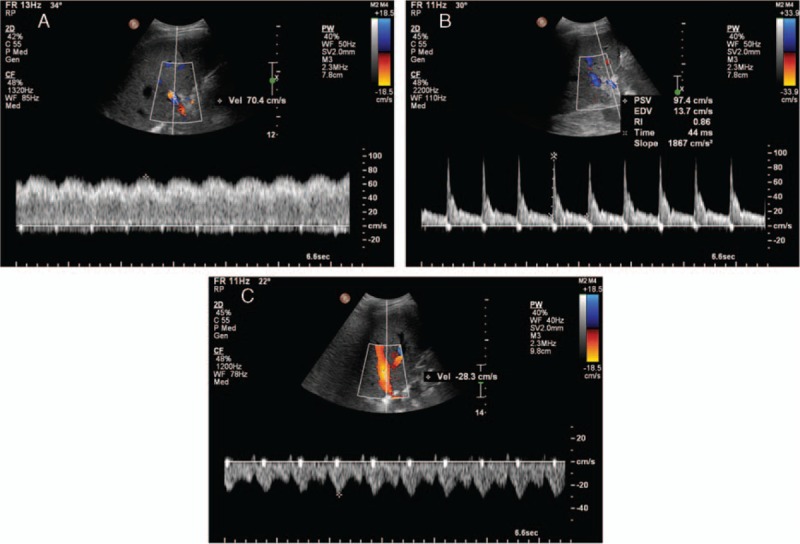
Measurement of PVV (A), PSV and EDV (B), and HVV (C) using spectral Doppler ultrasonography 1 day after living donor liver transplantation. RI is calculated by (PSV–EDV)/PSV. EDV = end diastolic velocity of the hepatic artery, HVV = peak hepatic venous flow velocity, PSV = peak systolic velocity of the hepatic artery, PVV = peak portal venous flow velocity, RI = resistive index.

One day after LDLT, peak portal venous flow velocity (PVV), peak systolic (PSV), and end diastolic velocities (EDV) of the hepatic artery, as well as peak hepatic venous flow velocity (HVV) were measured under spectral Doppler ultrasonography using a 1 to 5 MHz curved array transducer (C5-1, Philips Ultrasound, Bothell, WA) equipped to the diagnostic ultrasound system (iU22, Philips Ultrasound) (Fig. 1A–C). Next, we calculated the ratios of each velocity to 100 g of the initial GW which was obtained immediately after graft retrieval on the day of LDLT. Resistive index was calculated by dividing the difference between the PSV and EDV by the PSV ([PSV – EDV]/PSV). Before measurements, the patients were acclimatized on the bed for at least 15 minutes.

### 2.5 Measurement of the liver volume

Using the initial GW measured immediately after retrieval of the graft from donors, GRWR and GW/SLV were obtained. GRWR was calculated by dividing the GW by recipients’ body weight. As an index of expected size of the native liver in the recipient,^[13]^ SLV was calculated using the equation postulated by Urata et al^[14]^ as SLV (mL) = 706.2 × body surface area (m^2^) + 2.4, where body surface area (m^2^) = body weight (kg)^0.425^ × body height (cm)^0.725^ × 0.007184.

**Figure 2:**
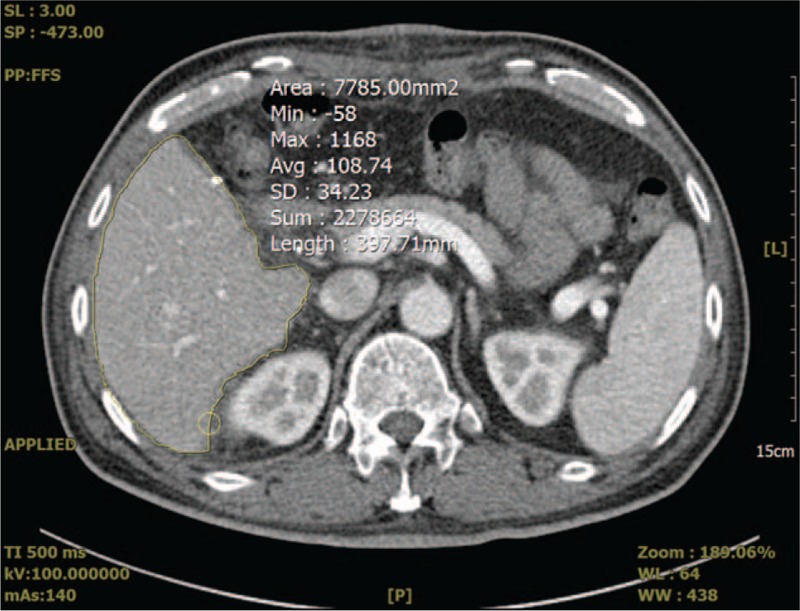
Measurement of one (7785 mm^3^) of the cross-sectional areas from the liver graft, which regenerated 2 weeks after living donor liver transplantation, by manual contour tracing of the hepatic contours. The volume of liver graft is calculated by multiplying sum of the cross-sectional areas of the liver graft by the slice thickness (3 mm).

To assess the postoperative regeneration of liver grafts, the regenerated liver volume was calculated by computed tomographic volumetry of multiple computed tomographic images obtained with a slice thickness of 3 mm at postoperative week 2 (Fig. 2). The calculated liver volume was substituted for actual liver weight, as the density of liver parenchyma is generally assumed to be 1.0 g/mL.^[15,16]^ In this study, the hepatic regeneration rate was defined as the ratio of regenerated volume measured using computed tomographic volumetry at postoperative week 2 to the initial GW.

### 2.6 Small-for-size syndrome

Patients were followed up until the end of study period (2 weeks after LDLT) and monitored for the development of small-for-size syndrome. This syndrome is defined as delayed functional hyperbilirubinemia: total serum bilirubin > 20 mg/dL for >7 consecutive days occurring after the 7th POD, excluding technical, immunological, and hepatitis factors.^[17]^

### 2.7 Sample size estimation

The primary end point of this study was hepatic regeneration rate at postoperative week 2. A total of 40 patients were required to achieve a statistical power of 80% with significance level of 0.05 (2-tailed) and drop-out rate of 10%, assuming that the alternative hypothesis of a 0.2 coefficient of determination between peak PVV/100 g of the initial GW (PVV/GW) on POD 1 and hepatic regeneration rate at postoperative week 2 is true.

### 2.8 Statistical analysis

The data are expressed as the mean ± standard deviation for normally distributed data and number of patients (percentage) for categorical data. The Kolmogorov-Smirnov and Shapiro-Wilk tests were used to assess the normality of distribution of the data. If at least one of the null hypotheses of the tests was not rejected, the assumption of normality was determined to be met. Simple linear regression analysis was used to investigate the relationship between hepatic hemodynamic parameter values and their values per 100 g of the initial GW on POD 1 and hepatic regeneration rate at postoperative week 2. All statistical analyses were performed using IBM SPSS Statistics software, version 19.0.0 (IBM Corp., Armonk, NY). A 2-tailed *P* < 0.05 was considered to be statistically significant.

## 3 Results

Four patients were excluded from this study because they underwent reinterventions during the study period including exploratory laparotomy for postoperative bleeding on POD 3 (n = 1) and spectral Doppler ultrasonography examinations more than 1 day after LDLT (n = 3). The mean initial GW was 681.7 g (Table 1). Although the GRWR and GW/SLV of 2 patients and GW/SLV of 1 patient were lower than the minimum size of a liver graft required to fulfill the metabolic demand of the recipient (0.8%^[1]^ and 35%^[18]^ for GRWR and GW/SLV, respectively), no patient developed small-for-size syndrome. The liver grafts regenerated to an average 170% of the initial GW (1123.3 ± 195.6 g), at postoperative week 2.

**Table 1:**
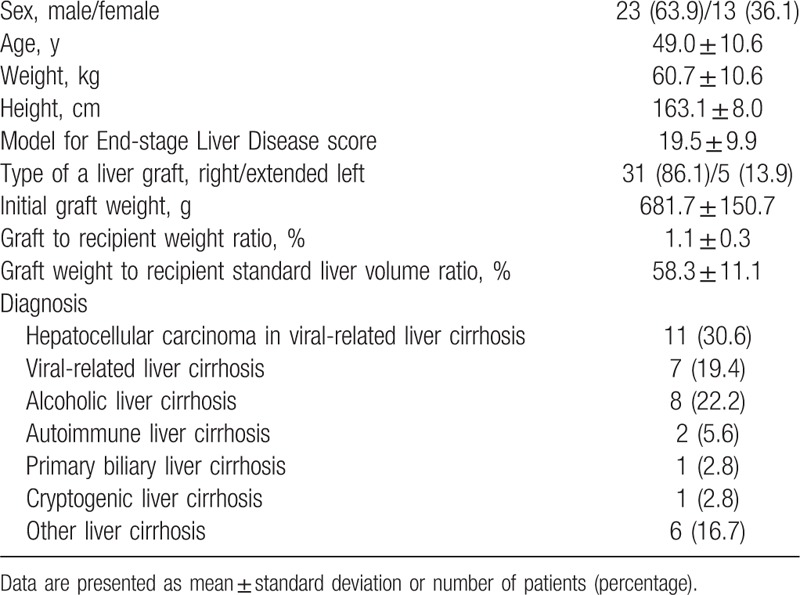
Demographic data.

**Figure 3:**
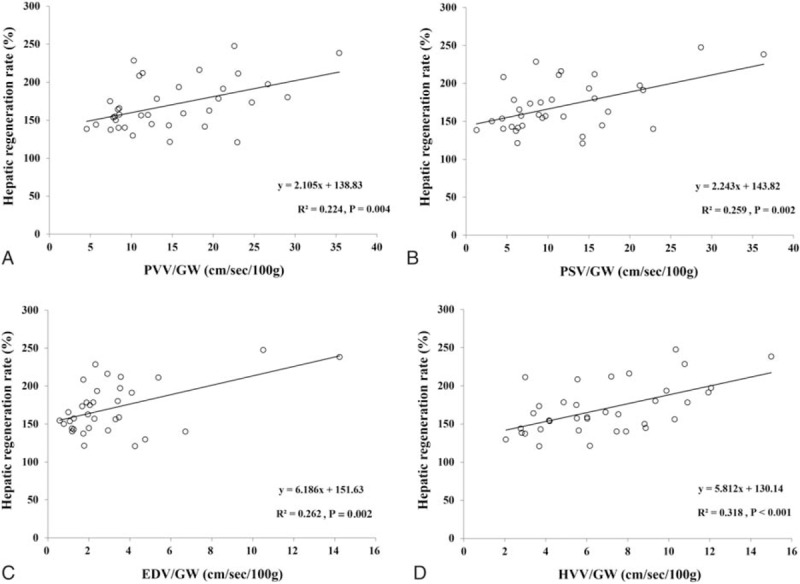
Positive correlations of PVV/GW (A), PSV/GW (B), EDV/GW (C), and HVV/GW (D) on postoperative day 1 with the hepatic regeneration rate at postoperative week 2. EDV/GW = end diastolic velocity of the hepatic artery per 100 g of the initial graft weight, HVV/GW = peak hepatic venous flow velocity per 100 g of the initial graft weight, PSV/GW = peak systolic velocity of the hepatic artery per 100 g of the initial graft weight, PVV/GW = peak portal venous flow velocity per 100 g of the initial graft weight. Initial graft weight: The liver graft weight measured immediately after its retrieval. Hepatic regeneration rate: The ratio of the liver graft weight measured at postoperative week 2 to the initial graft weight.

**Figure 4:**
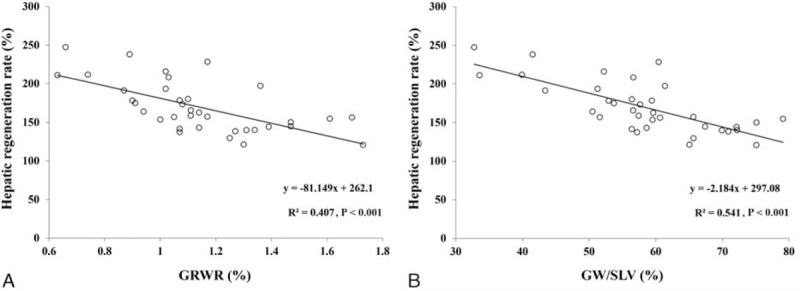
Negative correlations of GRWR (A) and GW/SLV (B) with the hepatic regeneration rate at postoperative week 2. GRWR = graft-to-recipient weight ratio representing the ratio of initial graft weight (the liver graft weight measured immediately after its retrieval) to recipient's weight, GW/SLV = graft-to-standard liver volume ratio representing the ratio of initial graft weight to standard liver volume (=706.2 × body surface area (m^2^) + 2.4 where body surface area (m^2^) = body weight (kg)^0.425^ × body height (cm)^0.725^ × 0.007184).^[14]^

There was no significant correlation between the hepatic hemodynamic parameters and hepatic regeneration rate. However, if the hepatic hemodynamic parameters were divided by 100 g of the initial GW, then PVV/GW, PSV/100 g of the initial GW (PSV/GW), EDV/100 g of the initial GW (EDV/GW), and HVV/100 g of the initial GW (HVV/GW) (Table 2) became positively correlated with the hepatic regeneration rate (*r*^2^ = 0.224, β_1_ = 2.105, and *P* = 0.004 for PVV/GW; *r*^2^ = 0.259, β_1_ = 2.243, and *P* = 0.002 for PSV/GW; *r*^2^ = 0.262, β_1_ = 6.186, and *P* = 0.002 for EDV/GW; *r*^2^ = 0.318, β_1_ = 5.812, and *P* < 0.001 for HVV/GW) (Fig. 3A–D). In addition, significant negative correlations were found between GRWR and hepatic regeneration rate (*r*^2^ = 0.407, β_1_ = −81.149, and *P* < 0.001) and between GW/SLV and hepatic regeneration rate (*r*^2^ = 0.541, β_1_ = –2.184, and *P* < 0.001) (Fig. 4A and B).

**Table 2:**
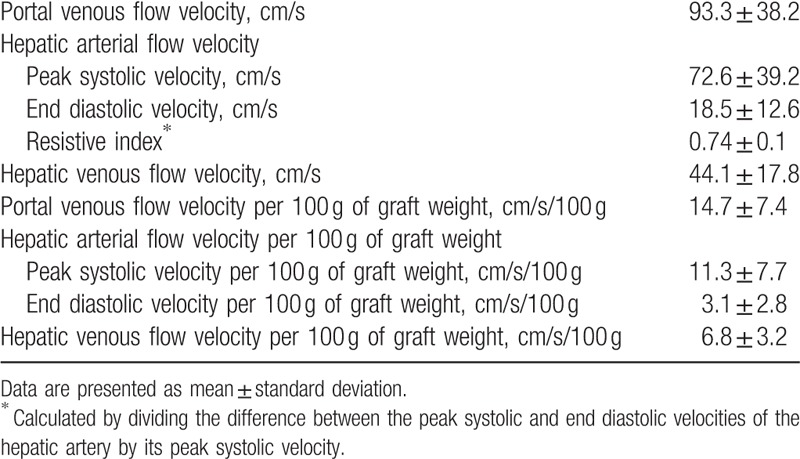
Hepatic hemodynamic parameters measured by spectral Doppler ultrasonography on postoperative day 1.

## 4 Discussion

In this study, the extent of graft hyperperfusion was evaluated by measuring the hepatic hemodynamic parameters per 100 g of the initial GW on POD 1, as well as the GRWR, and GW/SLV. We believe that the blood flow velocities of vessels in a liver graft represent their blood flow volume in the absence of vascular complications that significantly affect the patency of the blood flow. In addition, the blood flow volume per minute, which is calculated by multiplying the time-velocity integral per heart beat by heart rate and cross-sectional area of a vessel, is not accurate due to inter- and intraobserver variation producing high standard deviations.^[19–21]^ For these reasons, we preferred measuring the blood flow velocities of vessels to calculating their blood flow volume in order to assess the graft hyperperfusion. Therefore, higher velocities of inflow to the liver (portal vein and hepatic artery) represent more severe graft hyperperfusion. If inflow to the liver is constant, a small liver graft (with low GRWR or GW/SLV) would be hyperperfused. Based on the above concepts, positive correlations of PVV/GW, PSV/GW, and EDV/GW as well as negative correlations of GRWR and GW/SLV, with the hepatic regeneration rate indicate the contribution of hyperperfusion of liver graft to hepatic regeneration in the present study.

Our results showed a significant correlation between the PVV/GW on POD 1 and hepatic regeneration rate at postoperative week 2. However, there was no correlation between the PVV (not standardized by dividing with the GW) and hepatic regeneration rate. Likewise, a previous study reported a significant correlation between the PVV/GW on POD 1 and hepatic regeneration rate at postoperative week 1, but no correlation between PVV and hepatic regeneration rate.^[10]^ On the contrary, other studies found significant correlations between PVVs and various other indices representing hepatic regeneration obtained at different postoperative time points.^[2,9]^ However, these studies did not analyze data with PVV/GW. At this point, it is difficult to explain discrepancies in results between the studies because of differences in study designs.

The liver graft transplanted to a cirrhotic patient undergoes rapid hepatic regeneration that plateaus at GW/SLV of 100% at 2 weeks after LDLT, following which regeneration almost completely ceases.^[2]^ However, during the first 2 postoperative weeks, augmentation of graft volume might occur due to graft edema which prevents appropriate evaluation of hepatic regeneration.^[11]^ Therefore, the assessment of hepatic regeneration at postoperative week 2 seems reasonable to determine the extent of postoperative hepatic regeneration as early as possible in the absence of confounding effects caused by graft edema. Consequently, we evaluated hepatic regeneration at postoperative week 2. In contrast, the previous studies did not measure regenerated graft volume at postoperative week 2, preventing the possibility of a fair comparison.

Although several studies have demonstrated the beneficial effect of portal hyperperfusion on hepatic regeneration in LDLT recipients, these studies have certain limitations. In the study by Eguchi et al,^[2]^ several correlations of mean PVVs measured on POD 1, 7, or 28 with GW/SLV at postoperative week 1 or 2 and postoperative month 1 or 3 were found. However, the number of patients (n = 15) was too small to achieve sufficient statistical power. Furthermore, the etiology of liver disease was not consistent (4 patients with fulminant hepatic failure and 9 patients with liver cirrhosis). If fulminant hepatic failure develops in a patient with previously normal hepatic physiology, the amount of PVF to a liver graft would be significantly lower than that of a liver graft transplanted to a cirrhotic patient.^[2]^ Hence, the analysis of a patient group with mixed etiology is inappropriate. In addition, the slice width of their computed tomographic scan (7 mm) for the volumetric study was twice more than ours (3 mm). Another study, which showed the correlation of difference in PVF per 100 g of GW (PVF/GW) between recipient and donor with percent change in GW between time of graft harvest and postoperative month 2, enrolled 22 patients^[8]^ which is still smaller than number of patients (n = 36) in the present study. In addition, the accuracy of volumetric assessment could not be determined due to the absence of description of the slice width in magnetic resonance imaging. A more recent study reported the correlations of PVV/GW on POD 1 and PVF/GW on POD 1 and 5 or 6 with the hepatic regeneration rate at postoperative week 7.^[10]^ However, this study also included 3 patients with fulminant hepatic failure and assessed the hepatic regeneration rate earlier than 2 weeks after LDLT, when the effects of early graft edema on the liver graft are still prevalent. Lastly, Jiang et al's study,^[9]^ which showed the correlations of PVV and PVF on POD 1 and 3 with percent change in GW between preoperative period and POD 30, included only 18 patients. All the above studies standardized only the surgical technique without considering standardization of anesthetic management. To improve the design of our own study, we considered the limitations of previous studies, and estimated the sample size required to achieve a sufficient statistical power, tried to enroll patients with homogeneous etiology, used a uniform surgical and anesthesia protocol, and enhanced the accuracy of the computed tomographic volumetry with thinner slice width.

Due to the effects of hepatic arterial buffer response, which represents a reciprocal decrease in hepatic arterial blood flow in response to an increase in PVF on the liver graft,^[22]^ we did not expect that hepatic arterial blood flow would contribute to hepatic regeneration. However, we observed significant correlations between PSV/GW and EDV/GW with the hepatic regeneration rate. This unexpected effect of hepatic arterial blood flow might be attributed to the hepatic arterial buffer response blunted by the administration of prostaglandin E_1_ before the reperfusion of the hepatic artery.^[23]^

The relationship between hepatic venous flow or HVV and hepatic regeneration has not been extensively investigated. Although the grafts can regenerate successfully despite partial deprivation of venous drainage, hepatic venous outflow obstruction can cause graft congestion resulting in inappropriate regeneration of the affected area.^[24]^ Thus, the significant correlation between HVV/GW and hepatic regeneration in the present study strongly suggests that potent hepatic venous outflow that effectively delivers blood flow from the portal vein and hepatic artery can also contribute to hepatic regeneration. Therefore, to facilitate hepatic venous outflow, we performed a simplified one-orifice technique in the present study.^[12]^ However, until further evidence on the contribution of HVV to hepatic regeneration becomes available, this parameter should be used only as a reference and other factors which contribute to hepatic regeneration should be prioritized.

This study has several limitations as well. First, patients were followed up for only 2 weeks which prevented the assessment of long-term outcomes. 2nd, although it is known that graft edema resolves during the 2nd postoperative week,^[11]^ we cannot entirely exclude the possibility that graft edema persists after this time period and could have influenced our results. Third, despite continuous regeneration between the reperfusion of graft and POD 1, the initial GW was used to calculate the velocities per GW due to unavailability of the liver volume data on POD 1. Fourth, this study did not evaluate the relationship between hepatic blood flow and regeneration due to poor reproducibility of blood flow estimation using time-velocity integral and diameter of blood vessels.^[20]^ Lastly, multicollinearity caused by the high intercorrelations between the velocities, GRWR, and GW/SLV did not allow multivariate analysis in the present study.

In conclusion, we demonstrated positive correlations of the velocities of hepatic vessels per 100 g of GW measured on POD 1 with the hepatic regeneration rate measured 2 weeks after LDLT and negative correlations of GRWR and GW/SLV with the hepatic regeneration rate. In conclusion, graft hyperperfusion contributes to hepatic regeneration in the early postoperative period.
